# Smad4-dependent pathways control basement membrane deposition and endodermal cell migration at early stages of mouse development

**DOI:** 10.1186/1471-213X-9-54

**Published:** 2009-10-22

**Authors:** Ita Costello, Christine A Biondi, Jennifer M Taylor, Elizabeth K Bikoff, Elizabeth J Robertson

**Affiliations:** 1 Sir William Dunn School of Pathology, University of Oxford, South Parks Road, Oxford, OX1 3RE, UK; 2 Wellcome Trust Centre for Human Genetics, University of Oxford, Oxford, OX3 7BN, UK; 3 CSIRO Plant Industry, Black Mountain Laboratories, Clunies Ross Street, Black Mountain ACT 2601, Australia

## Abstract

**Background:**

Smad4 mutant embryos arrest shortly after implantation and display a characteristic shortened proximodistal axis, a significantly reduced epiblast, as well as a thickened visceral endoderm layer. Conditional rescue experiments demonstrate that bypassing the primary requirement for Smad4 in the extra-embryonic endoderm allows the epiblast to gastrulate. Smad4-independent TGF-β signals are thus sufficient to promote mesoderm formation and patterning. To further analyse essential Smad4 activities contributed by the extra-embryonic tissues, and characterise Smad4 dependent pathways in the early embryo, here we performed transcriptional profiling of Smad4 null embryonic stem (ES) cells and day 4 embryoid bodies (EBs).

**Results:**

Transcripts from wild-type versus Smad4 null ES cells and day 4 EBs were analysed using Illumina arrays. In addition to several known TGF-β/BMP target genes, we identified numerous Smad4-dependent transcripts that are mis-expressed in the mutants. As expected, mesodermal cell markers were dramatically down-regulated. We also observed an increase in non-canonical potency markers (*Pramel7*, *Tbx3*, *Zscan4*), germ cell markers (*Aire*, *Tuba3a*, *Dnmt3l*) as well as early endoderm markers (*Dpp4*, *H19*, *Dcn*). Additionally, expression of the extracellular matrix (ECM) remodelling enzymes *Mmp14 *and *Mmp9 *was decreased in Smad4 mutant ES and EB populations. These changes, in combination with increased levels of *laminin alpha1*, cause excessive basement membrane deposition. Similarly, in the context of the Smad4 null E6.5 embryos we observed an expanded basement membrane (BM) associated with the thickened endoderm layer.

**Conclusion:**

Smad4 functional loss results in a dramatic shift in gene expression patterns and in the endodermal cell lineage causes an excess deposition of, or an inability to breakdown and remodel, the underlying BM layer. These structural abnormalities probably disrupt reciprocal signalling between the epiblast and overlying visceral endoderm required for gastrulation.

## Background

Members of the TGF-β super-family of secreted growth factors activate a cell surface receptor complex comprised of two distinct transmembrane serine/threonine kinases that, upon ligand binding, phosphorylate members of the downstream receptor-associated Smads (R-Smads) (reviewed by [1]). The closely related R-Smads, Smad2 and Smad3 are phosphorylated in response to TGF-β s, Activin and Nodal signals. Smad1, Smad5 and Smad8 transmit BMP and GDF signals. The phosphorylated R-Smads in association with the common mediator Smad, Smad4, recruit additional cofactors to form higher order complexes that regulate target gene expression (reviewed by [2]). Smad4, originally discovered as a tumour suppressor gene, shares overall structural features with the R-Smads. However, its MH2 domain lacks the C-terminal SXS motif required for receptor-mediated phosphorylation. Smad4-RSmad complexes control a diverse array of biological processes, including cell proliferation, differentiation and cell survival during development and adult tissue homeostasis.

In the early embryo, reciprocal signalling between the epiblast, extra-embryonic ectoderm (ExE) and the overlying visceral endoderm (VE) is responsible for axis patterning and specification of the germ layers (reviewed by [3,4]). Members of the TGF-β/Nodal and BMP subfamilies act as morphogens that control cell differentiation in a concentration dependent manner. Nodal signals from the epiblast promote the formation of the distal visceral endoderm (DVE) [5,6]. This specialised cell population migrates anteriorly to become the anterior visceral endoderm (AVE) [7]. Expression of the Nodal antagonists *Lefty1 *and *Cer1 *by the AVE, is essential for patterning the underlying anterior epiblast [8]. On the posterior side of the embryo, BMP signals from the ExE together with Nodal signals from the epiblast promote primitive streak formation and mesoderm induction [9-12]. Continued Nodal signalling during gastrulation instructs epiblast cells passing through the anterior primitive streak to become definitive endoderm, prechordal plate, node and notochord [13]. Signalling via the BMP pathway is also crucial in early embryonic development (reviewed by [14,15]). Genetic studies demonstrate that activities of closely regulated R-Smads modulate dose-dependent Nodal and BMP signals in the early embryo [11,16].

*Smad4 *null embryos arrest shortly after implantation due to defects in the extra-embryonic lineages [17-19]. The mutants have a shortened proximodistal (P-D) axis, fail to acquire initial anterior-posterior (A-P) polarity, cannot gastrulate and are severely disorganised by E6.5. Early studies attributed the lethality to global proliferative defects [17,18] but conditional rescue experiments demonstrate that TGF-β signalling pathways in the embryo proper are surprisingly Smad4-independent [19]. Thus mutant epiblasts, in response to cues from wild-type extra-embryonic tissues, are able to gastrulate and generate diverse mesodermal derivatives [17,19], including the allantois, a rudimentary heart and mid-streak derivatives such as the somites and lateral plate mesoderm. Thus, early A-P axis formation and mesodermal patterning are unaffected. However, Smad4 is required for specification of the anterior primitive streak (APS) derivatives including the prechordal plate, node, notochord and definitive endoderm. Smad4 is also essential for BMP-dependent primordial germ cell (PGC) formation [19].

To investigate Smad4 requirements at early stages of embryonic development, here we exploited Smad4 null ES cells in transcriptional profiling experiments. We compared gene expression patterns in undifferentiated wild-type and mutant ES cells as well as embryoid bodies (EBs). The list of up- or down-regulated genes includes several previously described TGF-β/BMP/Smad targets. As expected, Smad4 mutant EBs show a marked decrease in gastrulation markers. The mutants display increased expression of several non-canonical potency markers, germ cell markers and early endoderm markers. Interestingly, Smad4 functional loss results in increased laminin expression and decreased expression of matrix metalloproteinases (Mmps). Mutant EBs display thickened endoderm, an expanded basement membrane layer and exhibit defective migratory properties. Collectively these results demonstrate that Smad4-dependent transcriptional regulation controls development of the extra-embryonic endoderm cell lineage.

## Results

### Transcriptional profiling of Smad4 null ES cells and EBs

To identify developmentally regulated transcripts that are potentially mis-regulated in the absence of Smad4, we analysed ES cells grown in the presence of LIF, or induced to differentiate as EBs in suspension culture. We compared mRNA expression patterns of wild-type and Smad4 null ES cells and day 4 EBs using the Illumina array platform. We utilised two independent 129S9/SvEvH wild-type (CCE/CCB) [20] and Smad4 null (FNN/BNN) ES cell lines [19]. After 4 days of suspension culture the outer cells of EBs are induced to differentiate to form a layer of primitive endoderm, while a sub-population of inner cells express nascent mesodermal markers. These cell aggregates closely resemble and share many characteristics of E6.5 embryos, the stage when growth defects become evident in Smad4 null mutants. Genes that displayed a 1.5 fold change and a statistical significance to p < 0.01 (corrected for multiple testing) were considered as differentially expressed. In Smad4 null ES cells, 243 probe sets detected transcripts up-regulated and 424 down-regulated, while in the EBs 674 and 464 probe sets detected increased and decreased transcript expression, respectively. Representative results are summarised in Table 1 and Table 2. The complete list of the mis-regulated genes is available in Additional Files 1 and 2. Data from the micro-arrays was validated using quantitative real-time PCR (Q-PCR) and analysed using the ΔΔCT method [21].

**Table 1:** Down-regulated genes in Smad4 null ES cells and/or day 4 embryoid bodies

**Down-regulated**				**Embryonic Stem Cell Array**			**Day 4 Embryoid Body Array**		
**Gene ID**	**Gene Description**	**RefSeq**	**Chr**	**ES Array Fold Change**	**Q-PCR data Fold Change**	**P-value**	**EB Array Fold Change**	**Q-PCR data Fold Change**	**P-value**

**Tgf-beta pathway**									

*Id1*	Inhibitor of DNA binding 1	NM_010495	2	9.2	8.43 ± 2.09	0.013	7.4	23.3 ± 4.14	0.0017
*Id2*	Inhibitor of DNA binding 2	NM_010496	12	3.8	7.23 ± 2.32	0.1193	1.7	n.d.	n.d.
*Id3*	Inhibitor of DNA binding 3	NM_008321	4	2.8	3.13 ± 0.72	0.0279	1.8	n.d.	n.d.
*Gdf1*	Growth differentiation factor 1	NM_008107	8	4.0	3.73 ± 0.18	<0.0001	1.75	4.01 ± 0.56	0.0018
*Lefty2 (Ebaf)*	Left-right determination factor 2	NM_177099	1	3.7	5.47 ± 1.18	0.0096	1.47	n.d.	n.d.
*Lefty1*	Left-right determination factor 1	NM_010094	1	1.6	1.77 ± 0.1	0.0004	-	n.d.	n.d.
*Tgfb2*	Transforming growth factor, beta 2	NM_009367	1	3.0	n.d.	n.d.	-	n.d.	n.d.
*Msx1*	Homeobox, msh-like 1	NM_010835	5	1.7	n.d.	n.d.	2.5	n.d.	n.d.

**Extracellular matrix related**									

*Mmp14*	Matrix metalloproteinase 14 (membrane-inserted)	NM_008608	14	4.5	5.44 ± 0.57	0.0002	3.2	5.16 ± 0.85	0.0028
*Mmp9*	Matrix metalloproteinase 9	NM_013599	2	1.6	2.97 ± 0.33	0.001	1.54	1.79 ± 0.28	0.092*
*Cd44*	CD44 antigen	NM_009851	2	2.7	7.25 ± 2.73	0.0625*	1.9	5.28 ± 1.62	0.0409
*Tgfb1i1 (Hic5)*	Transforming growth factor beta 1 induced transcript 1	NM_009365	7	2.8	7.39 ± 2.54	0.046	2.1	6.26 ± 1.95	0.0357

**Gastrulation markers**									

*Gsc*	Goosecoid homeobox	NM_010351	12	-	n.d.	n.d.	15	7.11 ± 0.79	< 0.0001
*Fgf8*	Fibroblast growth factor 8	NM_010205	19	2.0	n.d.	n.d.	14.1	13.85 ± 2.58	0.0006
*Sp5*	Trans-acting transcription factor 5	NM_022435	2	2.4	n.d.	n.d.	12	n.d.	n.d.
*T*	Brachyury	NM_009309	17	2.3	n.d.	n.d.	10.6	60.7 ± 8.43	0.0004
*Lhx1*	LIM homeobox protein 1	NM_008498	11	-	n.d.	n.d.	10.6	n.d.	n.d.
*MixL1*	Mix1 homeobox-like 1	NM_013729	1	-	n.d.	n.d.	6	13.98 ± 2.93	0.0046
*En1*	Engrailed 1	NM_010133	1	1.6	n.d.	n.d.	4.95	n.d.	n.d.
*Wnt8a*	Wingless-related MMTV integration site 8A	NM_009290	18	-	n.d.	n.d.	4.4	n.d.	n.d.
*Sox9*	SRY-box containing gene 9	NM_011448	11	-	n.d.	n.d.	3.5	n.d.	n.d.
*Fgf17*	Fibroblast growth factor 17	NM_008004	14	2.5	n.d.	n.d.	2.4	n.d.	n.d.
*Wnt9a*	Wingless-type MMTV integration site 9A	NM_139298	11	2.4	n.d.	n.d.	-	n.d.	n.d.
*Eomes*	Eomesodermin	NM_010136	9	-	n.d.	n.d.	2.1	8.03 ± 0.67	< 0.0001
*Chrd*	Chordin	NM_009893	16	-	n.d.	n.d.	1.97	n.d.	n.d.
*Cer1*	Cerberus 1 homolog	NM_009887	4	-	n.d.	n.d.	1.91	n.d.	n.d.
*Foxa2*	Forkhead box A2	NM_010446	2	-	n.d.	n.d.	1.8	8.98 ± 2.55	0.0216
*Fgf5*	Fibroblast growth factor 5	NM_010203	5	-	n.d.	n.d.	2.16	3.16 ± 0.14	< 0.0001

*Igf2*	Insulin-like growth factor 2	NM_010514	7	3.3	3.9 ± 0.72	0.0078	1.7	n.d.	n.d.

Micro-array and Q-PCR results are presented as fold change ratios relative to normalised wild-type values. The ES array results denote the average fold change of the two independent null cell lines. Genes with a 1.5 fold decrease were considered as differentially expressed. Q-PCR data is displayed as the mean ± standard error of the mean (SEM) of mRNA expression levels relative to *Hprt*. Genes validated by Q-PCR are significantly differentially expressed (p < 0.05), except for those indicated by an asterisk. n.d. validation not determined. A subset of differentially expressed transcripts is shown. * indicates higher than 0.05 P value for Q-PCR validation.

**Table 2:** Up-regulated genes in Smad4 null ES cells and/or day 4 embryoid bodies

**Up-regulated**				**Embryonic Stem Cell Array**			**Day 4 Embryoid Body Array**		
**Gene ID**	**Gene Description**	**RefSeq**	**Chr**	**ES Array Fold Change**	**Q-PCR data Fold Change**	**P-value**	**EB Array Fold Change**	**Q-PCR data Fold Change**	**P-value**

**Early ICM or ES markers**									

*Pramel7*	Preferentially expressed antigen in melanoma like 7	NM_178250	2	6.0	6.96 ± 1.09	0.002	-	n.d.	n.d.
*Calcoco2 (Ndp52l1)*	Calcium binding and coiled-coil domain 2	NM_029755	11	3.0	2.66 ± 0.39	0.0112	1.45	n.d.	n.d.
*Zscan4*	Zinc finger and SCAN domain containing 4C	NM_001013765	7	2.45	2.24 ± 0.32	0.0525*	2.1	2.94 ± 0.26	0.0002
*Magea8*	Melanoma antigen, family A, 8	NM_020020	X	2.325	10.7 ± 0.21	< 0.0001	-	n.d.	n.d.
*Tbx3*	T-box 3	NM_011535	5	3.0	2.06 ± 0.48	0.1673*	2.6	3.78 ± 1.08	0.0544*
*Pramel4*	Preferentially expressed antigen in melanoma like 4	NM_001001319	4	2.0	5.26 ± 0.58	0.0212	1.5	n.d.	n.d.

**Germ cell markers**									

*Tuba3a*	Tubulin, alpha 3A	NM_009446	6	4.8	5.76 ± 0.74	0.0013	4.1	5.46 ± 0.87	0.0024
*Dnmt3l*	DNA (cytosine-5-)-methyltransferase 3-like	NM_019448	10	-	n.d.	n.d.	3.7	4.43 ± 0.73	0.0038
*Smc1b (Smc1l2)*	Structural maintenance of chromosome 1b	NM_080470	15	5.7	n.d.	n.d.	2.8	n.d.	n.d.
*Dazl*	Deleted in azoospermia-like	NM_010021	17	3.1	n.d.	n.d.	1.5	n.d.	n.d.
*Aire*	Autoimmune regulator	NM_009646	10	2.8	5.11 ± 0.91	0.0056	4.1	5.03 ± 1.15	0.0139
*Wdr20b*	WD repeat domain 20b	NM_027614	12	2.6	n.d.	n.d.	1.45	n.d.	n.d.

**Endoderm markers**									

*Dpp4*	Dipeptidylpeptidase 4	NM_010074	2	2.8	n.d.	n.d.	3.8	2.52 ± 0.35	0.0111
*H19*	H19 fetal liver mRNA	NR_001592	7	2.1	1.58 ± 0.08	0.0049	2.7	3.81 ± 0.09	< 0.0001
*Dab2*	Disabled homolog 2	NM_023118	15	1.8	n.d.	n.d.	-	n.d.	n.d.
*Dcn*	Decorin	NM_007833	10	1.6	2.4 ± 0.27	0.0055	1.8	1.98 ± 0.56	0.1521*
*Lama1*	Laminin, alpha 1	NM_008480	17	-	n.d.	n.d.	3.9	n.d.	n.d.
*Hnf4a*	Hepatic nuclear factor 4, alpha	NM_008261	2	-	n.d.	n.d.	1.48	n.d.	n.d.
***Gata6***	**GATA binding protein 6**	NM_010258	18	**4.0**	**8.18 ± 1.71**	0.0005	-	n.d.	n.d.

Micro-array and Q-PCR results are presented as fold change ratios relative to normalised wild-type values. The ES array results denote the average fold change of the two independent null cell lines. Genes with a 1.5 fold decrease were considered as differentially expressed. Q-PCR data is displayed as the mean ± SEM of mRNA expression levels relative to *Hprt*. Genes validated by Q-PCR are significantly differentially expressed (p < 0.05), except for those indicated by an asterisk. Q-PCR validation for Gata6, indicated in bold, was performed using only the FNN Smad4 null ES cell line. A subset of up-regulated genes transcripts is shown. * indicates higher than 0.05 P value for Q-PCR validation.

**Figure 1:**
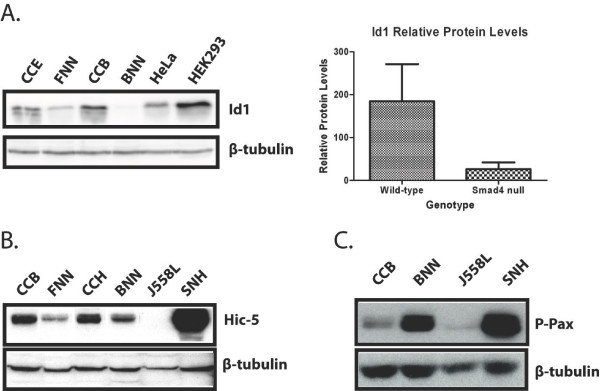
**Western blot analysis of candidate genes mis-regulated in Smad4 null ES cells**. **A**. Id1 expression levels are 7-fold lower in Smad4 null (FNN/BNN) cell lysates in comparison with wild-type (CCE/CCB) ES cells. Id1 protein levels were normalised to β-tubulin expression. HeLa, a human epithelial adenocarcinoma cell line, and the human embryonic kidney line, HEK293, were used as positive controls. **B**. Hic-5 protein levels are reduced in Smad4 null (FNN/BNN) in comparison with wild-type (CCB/CCH) ES cell lysates. **C**. Elevated phospho-Paxillin expression by Smad4 deficient ES cells. J558L, a myeloma cell line and embryonic fibroblasts, SNH, were used as negative and positive controls, respectively.

Numerous components of the TGF-β pathway are mis-regulated in Smad4 deficient cells including ligands (*Gdf1*, *Tgfb2*, *Lefty1/2*), as well as known target genes (*Id1/2/3 *and *Msx1*) (Table 1). The Id family of proteins regulate cell proliferation and differentiation in response to BMP signals [22,23]. *Id1*, *Id2 *and *Id3 *are selectively down-regulated in cardiac neural crest cells in conditional Smad4 mutants [24]. Up-regulated Id gene expression in response to BMP signalling is required to sustain self-renewal and pluripotency under serum-free conditions [25]. Here, loss of Smad4 function in ES cells and EBs results in reduced expression of *Id1*, *Id2 *and *Id3 *family members (Table 1). Western blots similarly reveal that Id1 protein levels are reduced by roughly 7-fold (Figure 1A).

Expression of *Msx1*, a Bmp responsive transcription factor, previously identified as a Bmp4 target in ES cells [26], is also down-regulated (Table 1;[19]). The Lefty proteins act as competitive inhibitors of Nodal and antagonise signalling via interactions with Nodal, as well as EGF-CFC co-receptors such as Cripto [27,28]. *Lefty 1 *and *Lefty 2 *expression is activated in response to Nodal/Activin signals [29,30]. Here we observe decreased *Lefty1 *and *Lefty2 *expression levels due to loss of Smad4 (Table 1). Collectively these results demonstrate that Smad4 controls expression of several known TGFβ/BMP target genes and suggest that this array platform offers a promising approach to characterise Smad4-dependent transcriptional networks that regulate early embryonic development.

### Increased steady state levels of phosphorylated receptor Smads

**Figure 2:**
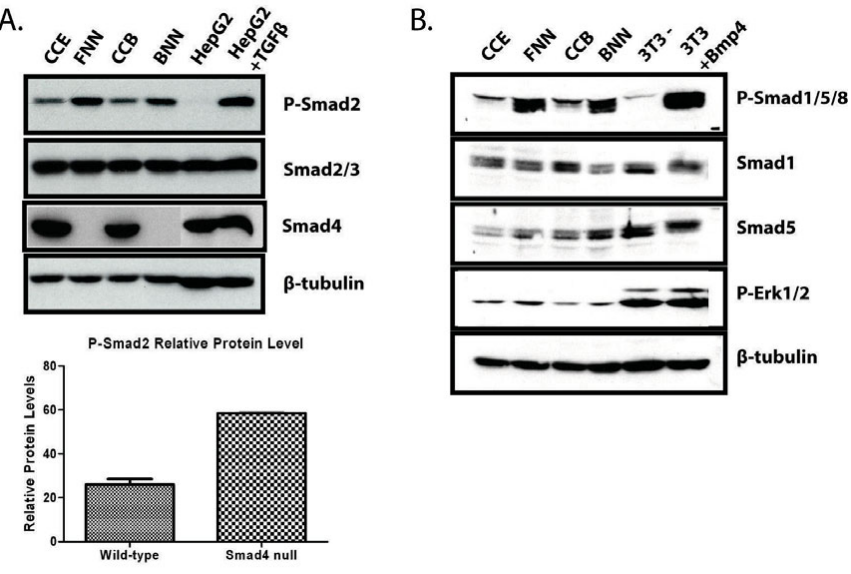
**Loss of Smad4 expression results in increased phosphorylation of R-Smads**. **A**. Western blots of lysates from wild-type (CCE & CCB) and Smad4 null ES cells (FNN & BNN) were probed with antibodies directed against R-Smads Smad2/3 or phospho-Smad2. β-tubulin acts as a loading control. Steady-state levels of Smad2/3 are unperturbed but P-Smad2 expression is strongly upregulated in Smad4 null ES cells. TGF-β stimulated HepG2 cells were included as a positive control. **B**. Similarly P-Smad1/5/8 levels are markedly enhanced in Smad4 null ES cells, wheras steady state levels of Smad1 and Smad5 are unaffected. Bmp4 treated NIH3T3 cells were included as a positive control. As judged by unchanged P-Erk1/2 levels loss of Smad4 function has no detectable effect on Erk signalling.

Smad4 deficient pancreatic carcinoma cell lines express increased levels of phosphorylated Smad2 (P-Smad2) [31]. However, in contrast, P-Smad2 levels were unaffected by Smad4 knock down in HaCaT cells [32]. We decided to compare basal TGF-β/BMP signalling in wild-type and Smad4 mutant ES cells. As shown in Figure 2, wild-type (CCE & CCB) and Smad4 null ES cells (FNN & BNN) express roughly equivalent steady-state levels of Smad2/3. However under normal culture conditions, in the absence of exogenous TGF-β ligands, Smad4 mutant ES cells constitutively express 2-2.5 fold more phospho-Smad2 in comparison with wild-type (Figure 2A). Similarly, loss of Smad4 has no effect on steady-state levels of effectors of the Bmp pathway, Smad1 and Smad5. However, we observed increased levels of P-Smad1/5/8 R-Smads (Figure 2B). Thus Smad4 loss results in increased phosphorylation of both categories of R-Smads. In contrast, phosphorylated Erk1/2 levels remain unchanged in the absence of Smad4 expression (Figure 2B).

### Mis-expression of non-canonical potency genes and germ cell markers

Smad4 null E6.5 embryos strongly express Oct4 [19]. Consistent with this, expression levels of the canonical core stem cell markers, *Oct4*, *Nanog *and *Sox2 *are unaffected in Smad4 mutant ES cells. However, numerous early embryonic and stem cell markers, including *Pramel7, Tbx3 *and *Zscan4*, are consistently up-regulated in Smad4 mutant ES cells (Table 2). *Pramel7 *is normally expressed at morula stages and is restricted to the inner cell mass of early blastocysts [33]. Pramel7 over-expression promotes LIF-independent self-renewal [33]. While transcripts are barely detectable in WT ES cells [34], *Pramel7 *expression increased roughly 6-fold in Smad4 mutant ES cells. Similarly, *Tbx3*, previously identified as a direct target of BMP Smads [35], is also up-regulated. *Tbx3 *plays an essential role in stem cell self-renewal and enforced expression represses mesodermal cell lineage commitment [36]. The zinc finger protein Zscan4 is normally expressed in late 2-cell embryos and a subpopulation of ES cells [37]. Previous experiments demonstrate *Zscan4 *is essential for pre-implantation development [37] and controls ES cell pluripotency [38]. Here we observed over 2-fold increased expression of *Zscan4 *in both mutant ES cells and EBs.

*Rhox5/Pem*, an X-linked homeodomain-containing gene, is up-regulated in mutant EBs. *Rhox5 *is normally expressed in morula and early blastocyst stage embryos, but shortly after implantation expression becomes restricted to extra-embryonic lineages, specifically the VE and extra-embryonic ectoderm (ExE) [39]. Interestingly, *Rhox5 *is normally expressed in ES cells, but is not detected in the primitive ectoderm [39] and *Rhox5 *over-expression inhibits ES cell differentiation [33,40]. Thus, Rhox5 function maintains early stem cell populations and promotes development of the extra-embryonic cell lineages. Collectively these results demonstrate that Smad4-dependent signalling regulates expression of potency genes.

Germ cell specification in response to BMP/Smad signals is strictly Smad4-dependent. Interestingly, expression of several germ cell markers was increased (Table 2). E2F6, which associates with polycomb group complexes, plays an important role in repressing meiosis-specific genes, including *Tuba3a *and *Smc1β *[41,42]. These E2F6 target genes are also mis-expressed in Smad4 deficient cells. These results strengthen the idea that Smad4-dependent signals regulate transcriptional networks upstream of early cell fate decisions.

### Smad4 mutant ES cells display increased expression of early endoderm markers

Smad4 loss results in increased expression of several endoderm specific markers (Table 2). For example, *Dpp4 *expression increased by roughly 3-fold. *Dpp4 *is normally activated in the VE shortly after implantation and is undetectable in the epiblast and undifferentiated wild-type ES cells [43]. Similarly, *Dab2 *is expressed in the VE and is required for surface sorting and positioning of endoderm cells [44,45]. *Dab2 *expression was increased in Smad4 mutant ES cells. *Dab2 *is a downstream target of *Gata6 *[46], a well-characterised endoderm marker [47,48]. Q-PCR shows roughly an 8 fold-increase in *Gata6 *levels in one of the Smad4 null ES cell lines (Table 2). *H19*, an imprinted gene that is normally expressed in the endodermal lineage [49], as well as *Decorin*, another endoderm marker, both show increased expression (Table 2). These results suggest that signalling cues responsible for guiding early endoderm development are substantially changed in the absence of Smad4.

### Dramatically decreased expression of gastrulation stage markers

Smad4 null embryos arrest at E6.5, fail to acquire initial anterior-posterior polarity and lack expression of nascent mesoderm markers. Previous studies described decreased expression of *T *(nascent mesoderm marker) and *Hnf4 *(primitive endoderm marker) at early stages of EB differentiation [17,19]. Conditional rescue experiments demonstrate that Smad4 is non-essential for mesoderm formation and patterning, but is required for development of the anterior primitive streak (APS) and its derivatives [19]. Here we detect a dramatic down-regulation of primitive streak markers in Smad4 null EBs (Table 1). Besides *T*, we also observe down-regulated expression of the Nodal targets (*Gsc *and *Foxa2*), as well as APS markers (*Gsc*, *Lhx1 *and *Foxa2*).

### Smad4-dependent pathways regulate expression of extra-cellular matrix components

Smad4 null ES cells and EBs also display decreased expression of several extracellular matrix (ECM) related genes (Table 1). Hic-5, also known as Tgfβ1i1, initially identified due to its TGF-β- and hydrogen peroxide-inducible expression [50], is a homolog of the multi-domain protein paxillin. Both Hic-5 and paxillin localise to focal adhesion sites and interact with the focal adhesion kinase (FAK) [51]. These membrane sub-compartments, where integrin clusters form, link the actin cytoskeleton with the ECM and are essential to mediate intracellular signalling. FAK phosphorylates paxillin to provide additional docking sites for downstream adaptor molecules that collectively regulate cell migration. In NIH 3T3 cells, Hic-5 competes with paxillin and inhibits paxillin phosphorylation [52]. Hic-5 also controls cell spreading and functions as a regulator of epithelial-to-mesenchymal transition (EMT) [52,53]. Here we observed decreased Hic-5 expression (Figure 1B) and conversely, as predicted, increased levels of phospho-paxillin in Smad4 null ES cells (Figure 1C).

### Smad4 loss results in defective endoderm migration during EB differentiation

**Figure 3:**
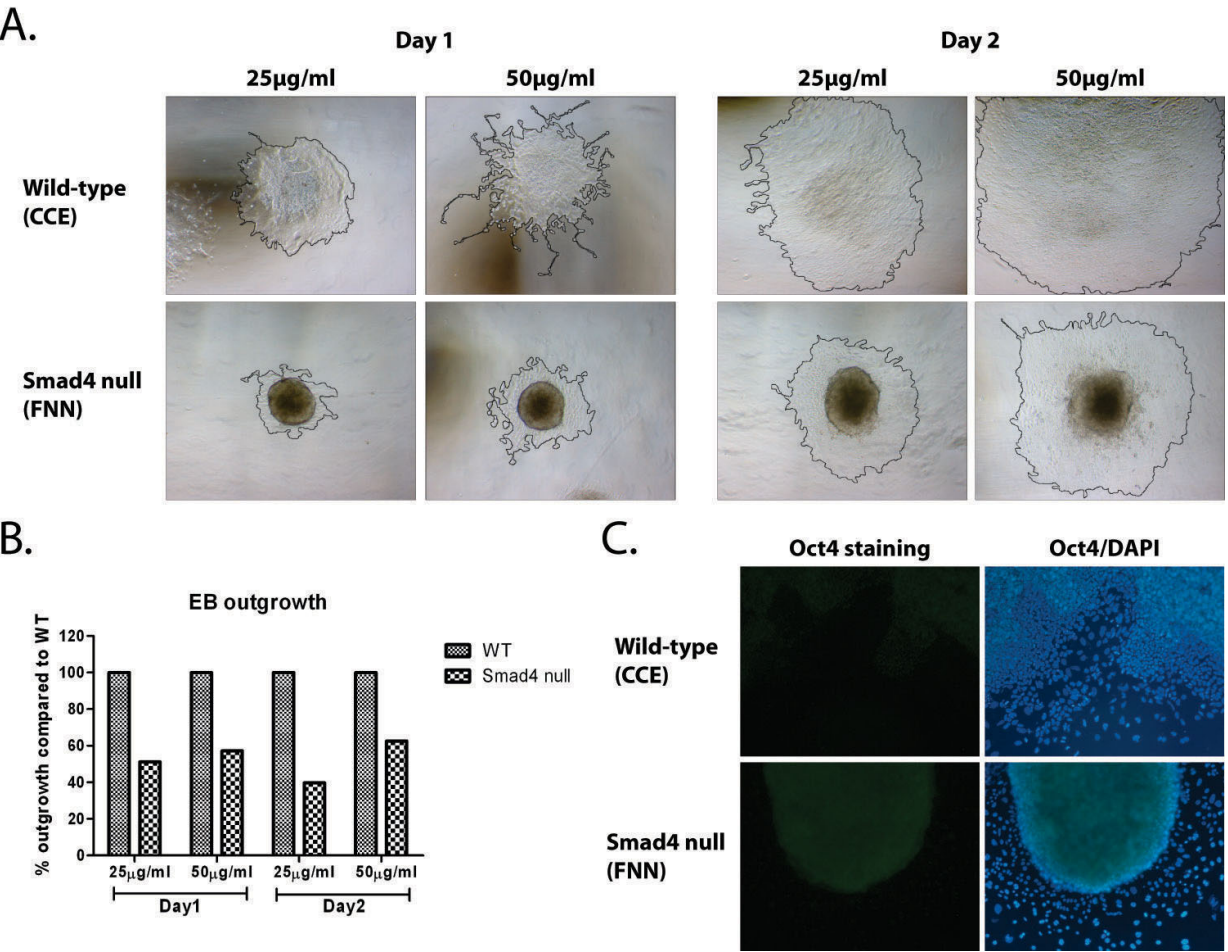
**Smad4 null embryoid bodies display defects in endoderm migration**. **A**. Day 4 wild-type and Smad4 null EBs were plated onto fibronectin-coated dishes and cultured for 2 days. At both fibronectin concentrations, outgrowth and migration were markedly reduced in Smad4 mutant EBs. **B**. The surface area of the outgrowths was compared using ImageJ. Smad4 null EB outgrowths, plotted as a percentage of the corresponding wild-type, show a reduction in the total surface area of outgrowth. **C**. Oct4 staining of EBs grown on 25 μg/ml fibronectin for 2 days. In contrast to barely detectable Oct4 levels in wild-type EB derivatives Smad4 null EBs retain robust expression.

Loss of Smad4 leads to increased expression of the ECM component *laminin alpha1 *(Table 2). ECM proteins regulate parietal endoderm differentiation and migration in EB outgrowth assays, in a fibronectin-dependent manner [54]. To directly test possibly impaired migratory abilities of Smad4 mutant endodermal derivatives, EBs grown in suspension were plated on fibronectin and allowed to attach and spread. Outgrowth and migration of Smad4 deficient EBs was dramatically reduced compared to wild-type (Figure 3A). Morphometric analysis revealed that migration decreased by roughly 50% (Figure 3B). Moreover, Smad4 mutant EB cultures grown in the absence of LIF for 6 days contain a substantial number of compact highly adherent cells expressing Oct4 (Figure 3C), a well-characterised pluripotency marker [55,56]. Thus, Smad4 is required to down-regulate expression of non-canonical early potency markers/transcription factors such as *Zscan4, Rhox5 *and *Tbx3 *(Table 2), while promoting the pathways responsible for endoderm formation.

### Smad4 functional loss causes defective ECM remodelling

Smad4 mutant ES cells and EBs also express decreased levels of matrix-metalloproteinases (Mmps), notably *Mmp14 *and *Mmp9 *(Table 1). Mmp14 is a membrane-tethered enzyme, while Mmp9 is secreted [57]. Mmp14 is considered as a master Mmp because it promotes activation of additional Mmps [58]. Mmp-mediated degradation of the ECM allows cells to migrate and also releases biologically active ligands, including TGF-βs, from the ECM [57]. Reduced *Mmp14 *expression has been described in Smad4 null neural crest derived cells [24], whereas, *Mmp9 *was previously identified as a Smad2/3 target gene in HaCaT cells [59].

**Figure 4:**
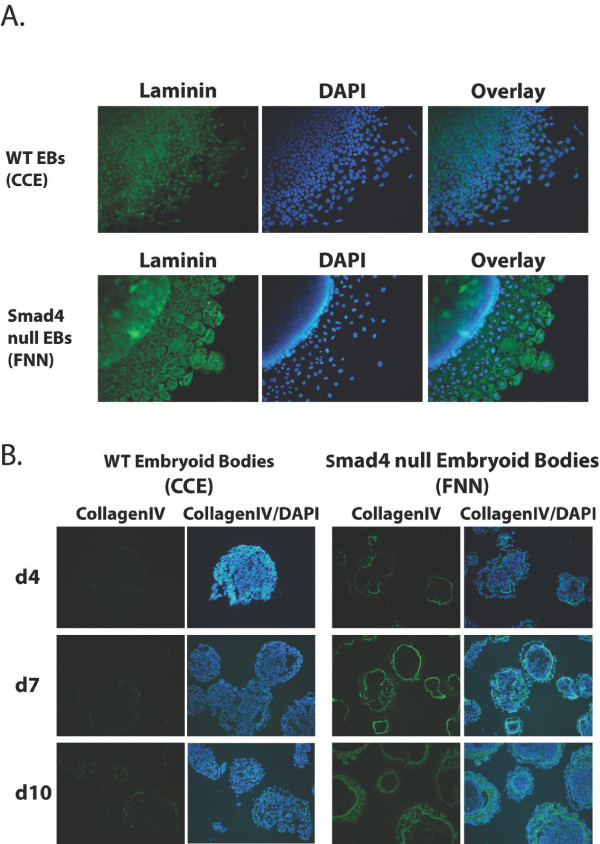
**Increased expression of basement membrane proteins by Smad4 mutant EB outgrowths**. **A**. Day 4 wild-type and Smad4 null EBs grown for 2 days on fibronectin-coated (25 μg/ml) dishes. Smad4 mutant EBs express increased levels of laminin. **B**. Cryosections of day 4, day 7 and day 10 suspension EBs stained for collagen IV. Smad4 mutant EBs display progressively increased basement membrane deposition beneath the outer endoderm layer.

**Figure 5:**
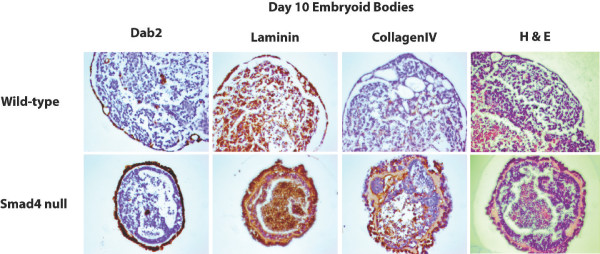
**Mature Smad4 mutant embryoid bodies display a thickened endoderm and expanded basement membrane**. The day 10 Smad4 null EBs strongly express Dab2. The thick extracellular matrix, detectable by hematoxylin and eosin (H&E) staining, stains positive for both laminin and collagenIV.

The present findings that Smad4 loss results in decreased *Mmp9 *and *Mmp14 *expression suggest that endodermal migration may potentially reflect an inability to breakdown and remodel the ECM. To evaluate this possibility, next we examined EB outgrowths for expression of the basement membrane components laminin and collagen IV. As shown in Figure 4A, Smad4 mutant outgrowths display a striking increase in extracellular deposition of basement membrane components. Laminin and collagen IV expression is readily detectable in wild-type cells, but is strongly up-regulated in the absence of Smad4 (Figure 4 & Additional file 3). *Laminin alpha1 *transcripts are also up-regulated (Table 2). Increased production of ECM proteins, in combination with reduced levels of ECM degrading enzymes, thus results in enhanced deposition of basement membrane components and decreased migration across the fibronectin substrate. Similarly, increased levels of laminin and collagen IV expression and hence an enlarged basement membrane underlying the outer layer of primitive endoderm was detected in Smad4 mutant EBs (Figure 4B & Figure 5). Additionally, the outer endoderm layer was thicker and strongly positive for Dab2 and the mutant EBs were smaller than the WT controls overall (Figure 5). Thus the differences initially detectable in early day 4 EBs continued throughout the differentiation process (Figure 4B & Figure 5). The excess production of *laminin alpha1 *in combination with reduced expression of the ECM degrading enzymes *Mmp14 *and *Mmp9 *(Table 1) disrupts migration and signalling by the Smad4 mutant endoderm.

### Smad4 mutant embryos contain an excessive embryonic basement membrane

**Figure 6:**
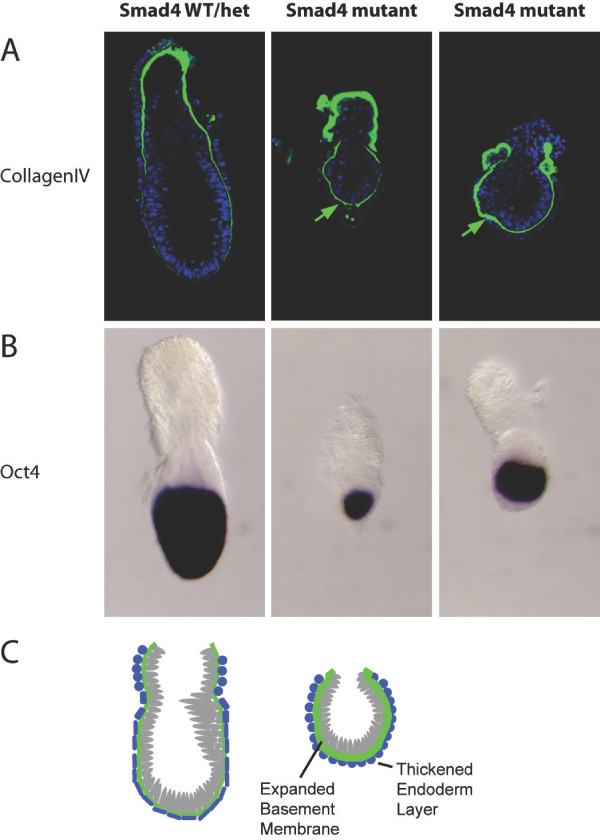
**Visualising defective endoderm in E6.5 Smad4 mutant embryos**. **A**. Collagen IV staining of the thickened basement membrane (green arrows) in Smad4 null embryos. Blue, DAPI nuclear staining. **B**. The Smad4 mutant epiblast strongly expresses Oct4 and is significantly reduced in size. **C**. Schematic of wild-type versus Smad4 null E6.0-E6.5 embryos. Blue corresponds to endoderm cells, grey represens the epiblast and green indicates the basement membrane layer.

To examine whether similar changes contribute to the mutant phenotype *in vivo*, we analysed Smad4 null embryos. As expected, the VE is distinctly thicker and the epiblast is significantly reduced in embryos lacking Smad4 (Figure 6) [17-19]. Previously published H&E sections clearly show a thickened single endoderm layer [19]. Interestingly as shown in Figure 6A we observe a marked increase in collagen IV staining between the epiblast and visceral endoderm layer. As shown above, mutant endodermal cell populations display increased deposition of basement membrane that probably leads to a complete block of reciprocal signalling between the extra-embryonic endoderm and epiblast.

## Discussion

Mis-regulated genes identified here in Smad4 mutant ES cells and those previously characterised in screens analysing Smad4 targets in tumour cell lines show surprisingly little overlap [32,60,61]. Besides technical issues, such as the use of different array platforms, this difference probably also reflects cell type specific gene expression patterns. We employed *Smad4 *genetically null cells, whereas previous studies analysed the consequences of knock-down of *Smad4 *using RNAi or profiled tumour cell lines, in all likelihood carrying multiple mutations that could potentially complicate the analysis. *Smad4 *is broadly expressed in embryonic and adult tissues. Nonetheless, cellular responses activated by TGF-β/Nodal/BMP signalling pathways are remarkably diverse. Thus it seems likely that Smad4-dependent targets will be influenced by cell-type specific partnerships and are highly context dependent.

**Figure 7:**
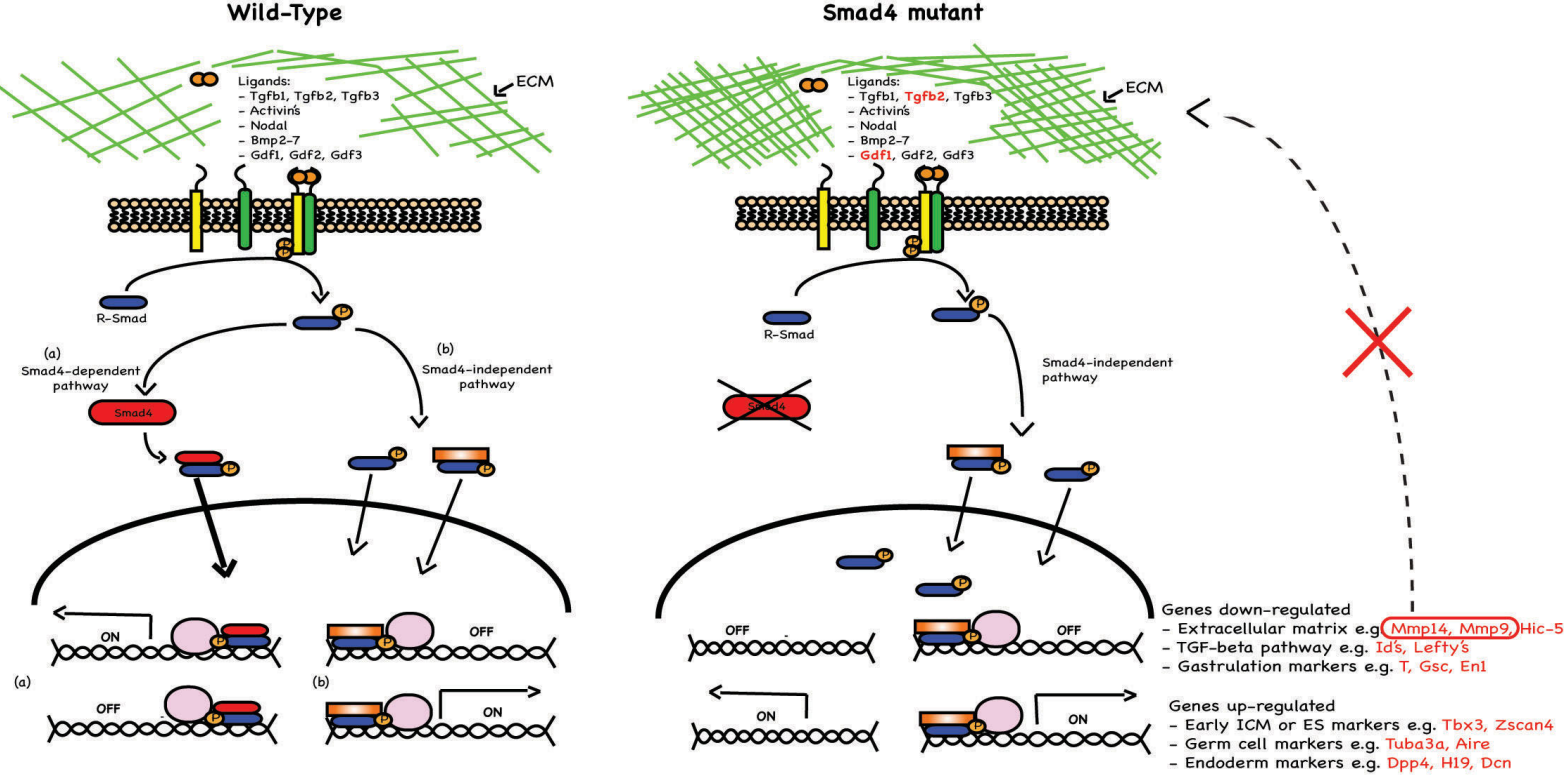
**Smad4 controls transcriptional progammes in the early embryo**. Loss of Smad4 is associated with increased levels of phosphorylated R-Smads and a dramatic shift in gene expression patterns. In wild-type cells TGF-β/BMP/Smad pathways regulate target gene expression via (a) Smad4-dependent or (b) Smad4-independent mechanisms. In the absence of Smad4, phosphorylated R-Smads are efficiently translocated to the nucleus on their own or by interacting with another partner (indicated as an orange box). The pink circles indicate transcriptional partners required for activation or repression of genes. Mis-regulated genes in Smad4 mutant cells potentially reflect the loss of Smad4-dependent pathways or could also be due to increased Smad4-independent signalling. In Smad4 mutant cells, genes in red are mis-regulated resulting in defective endoderm differentiation and massive deposition of BM components (indicated by green mesh). In *Drosophila*, components of the BM, namely Collagen IV, are known to modulate BMP signalling in the extracellular space during development [89]. Overall the present results demonstrate TGF-β/BMP/Smad signals control expression of the extracellular matrix and reciprocally, BM components fine tune Smad signalling.

Previous studies demonstrate that growth characteristics of Smad4 null ES cells are indistinguishable from wild-type [17,62]. As judged by Smad2 phosphorylation, TGF-β/Nodal signalling is constitutively active in undifferentiated ES cells ([63] & this study), but its role in promoting ES cell self-renewal remains unclear [63,64]. BMP signalling activates the Id family of target genes and is required to sustain self-renewal and pluripotency of ES cells [25]. Smad4 nucleocytoplasmic shuttling is not required for R-Smad phosphorylation or nuclear localisation [65]. R-Smad dephosphorylation and nuclear export is thought to be required for optimal TGF-β signalling [66,67]. Here we demonstrate that Smad4 loss leads to increased steady state levels of both BMP and TGF-β phosphorylated R-Smads (Figure 7). Enhanced R-Smad phosphorylation levels potentially reflect decreased dephosphorylation and/or nuclear export [66,67]. In the absence of Smad4, R-Smads may be less efficiently recognised by C-terminal phosphatases and/or actively retained in the nucleus.

Another possibility is that increased levels of phosphorylated R-Smads may be caused by reduced expression of Nodal antagonists, namely *Lefty1 *and *Lefty2 *[27,28]. The lack of negative feedback regulation may dramatically shift gene expression patterns. Smad4 conditional deletion in cardiomyocytes results in increased phosphorylation of Erk1/2 [68] and activation of the MAPK pathway probably contributes to defective heart development. Erk1/2 signalling has also been implicated in controlling the transition of ES cells from self-renewal to differentiation [69]. Here, we observe loss of Smad4 in ES cells has no effect on phospho-Erk1/2 activity. Increased R-Smad phosphorylation in the absence of enhanced MAPK signalling may also contribute to the changes in transcriptional profiles.

As expected Smad4 mutant ES cells and EBs display dramatically reduced levels of nascent mesoderm markers (e.g. *T*, *Mixl1*, *Gsc*). Additionally, loss of Smad4-dependent signals results in increased expression of non-canonical stem cell genes, including *Pramel7*, *Zscan4 *and *Tbx3*. The precise roles played by these potency genes, (notably *Calcoco2*, *Pramel4*, *Zscan4*), remains ill defined. *Pramel4*, *Pramel7 *and *Calcoco2 *were initially identified as *Oct-4 *related genes [70]. *Pramel7 *is normally expressed in the compacted morula and the inner cell mass (ICM) of the early blastocyst and *Pramel7 *over-expression results in LIF-independent self-renewal [33].

Only one *ZSCAN4 *gene has been identified in humans, while nine paralogous *Zscan4 *genes are present in the mouse genome [37]. Of these, six are known to be expressed and three of these, namely *Zscan4c*, *Zscan4d *and *Zscan4f*, encode highly similar proteins [37]. *Zscan4d *is an abundant transcript at the 2-cell stage but its expression is rapidly turned off. Expression of *Zscan4c*, and to a lesser extent *Zscan4f*, is up-regulated in blastocyst outgrowths and is detectable in a mosaic fashion in ES cultures [37]. RNAi knock-down experiments demonstrate that *Zscan4 *is essential for pre-implantation development [37].

*Tbx3 *is also required for ES cell proliferation. Enforced expression results in LIF-independent self-renewal and is sufficient to repress mesodermal commitment [36]. Similarly, *Rhox5 *over-expression maintains ES cell self-renewal in the absence of LIF [33] and inhibits EB differentiation [40]. *Rhox5 *is also expressed in the primitive endoderm but its role *in vivo *remains unknown. Interestingly, expression of the autoimmune regulator *Aire *is also up-regulated in Smad4 mutant cells. *Aire *is normally expressed in medullary thymic epithelial cells (MTECs) and in the testis [71,72]. *Aire *expression by MTECs induces expression of *Nanog*, *Oct4*, and *Sox2*, and is required for promiscuous expression of tissue-restricted antigens [71,73]. These activities are required for imposing central tolerance and controlling autoimmunity. Increased *Aire *expression may similarly contribute to promiscuous gene expression patterns described here in Smad4 mutant ES cells.

Smad4 mutant ES cells also up-regulate expression of *Dab2*, *Rhox5 *and *Hnf4a*, which are normally expressed in the early primitive endoderm [74]. The relatively immature Smad4 mutant endoderm cells may fail to progress towards a more differentiated VE state. Consistent with this idea, the endoderm formed by mutant EBs displays a distinctive more rounded thicker morphology, similar to that present in mutant embryos. Additionally, marker genes normally present in mature VE such as *Fgf8*, *Foxa2*, *Cer1*, *Gsc *and *Lhx1 *are markedly down-regulated. Another possibility is that these genes are under-represented due to the block in A-P patterning and/or the failure to induce mesodermal and definitive endoderm cell lineages. These observations reinforce the idea that Smad4-dependent signals are essential in the early primitive endoderm to promote reciprocal signalling and pattern the underlying epiblast.

During mouse embryogenesis, the primitive endoderm generates a layer of BM between the VE and the epiblast (embryonic BM), while the highly secretory parietal endoderm (PE) cell population, in conjunction with the trophectoderm, is responsible for producing Reichert's membrane, the protective layer between the embryo and the maternal uterine environment. The BM overlying the embryo polarises the epiblast and stimulates cavitation [75]. Numerous mutations that disrupt basement membrane synthesis cause early post-implantation lethality [76-78]. Mmps play important roles in ECM remodelling, however their functional contributions to early embryonic development remains ill defined. It has been proposed that Mmps synthesised by the trophectoderm promote implantation of the embryo into the uterine wall [79]. *Mmp9 *and *Mmp2 *are known to be expressed by ICM outgrowths and cultured PE-like cells [80,81]. However, none of the Mmp loss of function mutations generated to-date disrupt early development. In all likelihood the failure to observe embryonic phenotypes simply reflects the extensive overlapping expression patterns and functional redundancy among close family members [57,82].

Numerous ECM-related genes, including *PAI-1*, *fibronectin *and pro-collagens, are controlled by Smad4-independent signals [32,62]. The present experiments demonstrate for the first time that remodelling ECM components in the early embryo requires Smad4-dependent signals (Figure 7). Besides breaking down the ECM, Mmps can also release cell surface associated growth factors from the ECM. In particular, Mmp2 and Mmp9 release biologically active TGF-β [83]. Cd44 and TGF-β are known Mmp substrates [83,84] and intriguingly both *Cd44 *and *Tgf-β2 *are mis-regulated in Smad4 mutant ES cells.

Reduced *Mmp14 *and *Mmp9 *expression in combination with up-regulated laminin production, leads to excessive BM deposition and results in endoderm migration defects in EB outgrowth assays. Interestingly, previous studies demonstrate that endodermal derivatives of ES cells, with a constitutively active Akt/PKB pathway, similarly produce excess BM underlying the endoderm layer, due to a massive increase in laminin-1 and collagen IV synthesis [85]. We speculate that cross-talk between TGF-β and Akt/PKB signalling pathways may cooperatively regulate expression and remodelling of ECM components in the early embryo.

The present results demonstrate that Smad4 mutant endodermal derivatives display defective migration in EB outgrowth assays. Similarly, Smad4 regulates TGF-β induced cell migration in keratinocytes and pancreatic tumour cells [32]. The epithelial-mesenchymal transitions essential in the early embryo and those associated with tumour progression exhibit striking similarities. Surprisingly little is known about TGF-β/Smad signals required for guiding endodermal cell migration at early stages of development. Smad4 null blastocysts display defective outgrowth [18] but cell migration and proliferative capabilities are tightly coupled, making it difficult to distinguish whether Smad4 loss results in migration defects per se or can be explained due to decreased cell proliferation. Results of the EB outgrowth assays presented here clearly demonstrate that Smad4 regulates endoderm migratory capabilities *in vitro*. However, formation of the parietal endoderm is not impaired in Smad4 mutant embryos. Thus synthesis of Reichert's membrane forms normally despite the small size of the embryo [18]. Rather, loss of Smad function selectively disrupts VE development, probably due to impaired reciprocal signalling between the VE and the epiblast. Nodal signalling from the epiblast induces the AVE in the overlying endoderm via a Smad2 dependent pathway [5]. In turn, activation of key targets in the AVE including *Foxa2*, *Lhx1*, *Cer1 *and *Lefty1 *provide important signalling cues for patterning the epiblast (reviewed by [4]). The present experiments demonstrate that Smad4 mutant EBs show reduced expression of *Cer1*, a negative regulator of TGF-β/BMP signalling, as well as *Foxa2 *and *Lhx1 *(Table 1). Excess basement membrane produced by Smad4 mutant endoderm likely acts as a reservoir trap for biologically active ligands and causes defective cell-cell communication between the epiblast, visceral endoderm and extra-embryonic ectoderm.

## Conclusion

Our transcriptional profiling experiments have identified numerous genes that are differentially expressed in Smad4 mutant ES cells and EBs. Analysis of the gene list provides new insights into the tissue defects in Smad4 mutant embryos. The failure to down-regulate the non-canonical potency markers causes defective endodermal cell lineage commitment. Developmental arrest is associated with excess basement membrane, resulting from increased production of the ECM components and decreased expression of Mmps required for remodelling (Figure 7). The thickened BM disrupts reciprocal signalling between the VE and epiblast. Hence, deregulated expression of Smad4 target genes in the primitive endoderm results in the inability to form mesoderm and definitive endoderm.

## Methods

### RNA analysis

Wild-type (CCE & CCB) and *Smad4 *null (FNN & BNN) 129S9/SvEvH embryonic stem cell lines adapted to grow under feeder independent conditions were routinely expanded on gelatin coated tissue culture dishes in DMEM plus 15% FCS with 1000 units/ml recombinant LIF (Millipore). Embryoid bodies generated as described [86] were harvested at day 4 of differentiation. For RNA isolation, ES cells were seeded at 1 × 10^6 ^cells per 6 cm dish and harvested the next day at 70-80% confluency. EBs were harvested by centrifugation. Lysates prepared in RLT buffer (Qiagen) were removed by scraping and applied to a QIAshredder (Qiagen). RNA from both ES cells and EBs was isolated using an RNeasy kit (Qiagen) accordingly to the manufacturer's instructions.

### Array

The ES cell Array data was generated in two phases. Intially two sets of three technical replicates from wild-type CCE and Smad4 null FNN ES cell lines were collected one passage apart. In the second phase three independent technical replicates collected from wild-type CCB and Smad4 null BNN ES cell lines. Each dataset was analysed in an identical manner. The EB array was performed in one phase using two technical replicates of each of all of the above four cell lines. The Illumina WG-6 Mouse v1 Sentrix BeadArray contains 46,630 unique probe sequences targetting 34,784 unique RefSeq accession identifiers and 33,720 gene symbols. Samples were arranged randomly with respect to the 6 array positions within each BeadArray. Arrays were scanned according to manufacturer's instructions and raw intensity data extracted for each probe type using the software BeadStudio version 3.0.14. Raw signals were subtracted for background intensities and imported into R statistical scripting environment . Data was transformed using a variance stabilisation algorithm as implement in the BioConductor  package, vsn [87] and quantile normalised. Unsupervised analysis consisted of hierarchical clustering using euclidean distance and complete linkage. Differentially expressed probes were identified using the bioconductor package limma using a univariate linear model containing a genotype effect. For phase 1 an additional covariate was added for passage groups. P values were adjusted for multiple testing using the Benjamini-Hochberg false discovery rate. Probes were considered differentially expressed relative to genotype if they meet a P < 0.01 significance after adjustment for multiple testing.

Probe identifiers were fully annotated using the Mouse-6_v1_1_11234304_A array manifest. Gene ontology enrichment analysis was performed using the online DAVID bioinformatics resources  which uses a modified Fisher Exact test to ascertain whether certain gene functions are enriched in a specific gene list above what would be expected through random sampling of the array. The background frequencies of gene ontology terms were generated using all identifiers represented on the Illumina array.

### Q-PCR Validation

Array data was validated by quantitative PCR analysis using a Corbett Rotagene 3000. cDNA was generated using the Superscript III Kit (Invitrogen) with oligo-dT primers. One tenth of the product was amplified in a 15 μl SYBRGreen PCR reaction (Qiagen). Cycling conditions incorporated an initial denaturation step of 15 min at 94°C; 2 × (30 s at 94°C, 1 min at 72°C and 1 min 30 s at 72°C); 2 × (30 s at 94°C, 1 min at 70°C and 1 min 30 s at 72°C); 30 × (30 s at 94°C, 1 min at 55-62°C and 1 min 30 s at 72°C) followed by a final extension cycle of 5 min at 72°C. Primer sequences and annealing temperatures are provided in Additional File 4. Quantification of fold-changes initially compared both biological replicates and was further validated using additional technical replicates. Relative gene expression was deduced using the ΔΔCt method [21] in comparison with *Hprt *as the reference. Ct values of each qPCR reaction were normalised with the respective Ct values of the *Hprt*. The fold change was calculated using the formula: Fold change = 2 ^-(ΔCtgene1-ΔCtgene2)^. The resulting fold change is expressed as mean ± SEM. Statistical analysis was performed using the Prism5 statistic package and the Students T-test.

### Western blot analysis

Cell lysates prepared from *Smad4*^+/+ ^(CCE, CCB) and *Smad4*^*N*/*N *^(FNN, BNN) ES cell lines using RIPA buffer were subjected to SDS-PAGE and transferred onto PVDF membranes. Membranes were blocked with 5% milk in TBS-T then incubated in primary antibodies overnight including rabbit anti-phospho-Smad2 (Cell Signalling 3101L; 1:1000), mouse anti-Smad2/3 (Transduction 610843; 1:500), rabbit anti-β-tubulin (Santa Cruz sc-9104; 1:1000), rabbit anti-Id1 (Santa Cruz sc-488; 1:500), rabbit anti-phospho-Smad1/5/8 (Upstate AB3848; 1:1,000). Mouse anti-Smad1 (Santa Cruz sc-7965; 1:200), goat anti-Smad5 (Santa Cruz sc-7443; 1:200), rabbit anti-phospho p44/42 MAP kinase (Erk) (Cell Signalling 9101; 1:1000), rabbit anti-phospho-paxillin (Tyr 118) (Cell Signaling 2541; 1:1,000), mouse anti-Hic5 (BD Transduction 611164; 1:250). Secondary antibodies were donkey anti-rabbit HRP (Amersham NA934V; 1:2000) and sheep anti-mouse-HRP (Amersham NA931V; 1:2000). Blots were developed by chemiluminescence using ECL plus (Amersham) and quantified using a Bio-Rad GelDoc and the Quantity one software.

### Embryoid body outgrowth and migration assays

Wild-type and *Smad4 *null d4 EBs were plated in Lab-Tek chamber glass slides (Nunc) previously coated 25-50 μg/ml with fibronectin (Sigma), cultured for 2 days, rinsed with PBS, fixed with 4% paraformaldehyde, washed in PBS and then permeabilized with 0.3% Triton X-100-PBS and blocked in 10% FCS, 0.3% BSA, 0.3% Triton X-100 in PBS for 30 min at room temperature. Slides were then incubated with primary antibodies as follows, anti-laminin (Sigma, L-9393; 1:200); anti-collagenIV (Chemicon, AB756P; 1:100) anti-Oct4 (Santa Cruz sc-8628; 1:200). Slides were incubated with either a goat anti-rabbit IgG or donkey anti-goat IgG secondary antibody (Alexa Fluor-488, Molecular Probes; 1in 500) and mounted with Vectashield mountant containing the nuclear counterstain 4',6-diamidino-2-phenylindole (DAPI) (Vector Labs H-1200). Fluorescent images were captured with a Zeiss epifluorescence microscope. Morphometric analysis was carried out using the ImageJ program.

### Cryo-sectioning, immunocytochemistry and immunohistochemistry

EBs were washed with PBS, fixed in 4% paraformaldehyde for 30 minutes at room temperature (RT), washed in PBS, transferred into 7.5% sucrose in PBS for 3 hrs at RT and then immersed in 15% sucrose in PBS overnight at 4°C. The sucrose solution was removed, replaced with Tissue-Tek O.C.T. compound and samples transferred into a cryo mould and frozen in an iso-pentane bath on dry ice. 7 μm cryosections were collected onto gelatin coated positively charged Superfrost glass slides and were stained with primary antibodies: anti-laminin (Sigma, L-9393; 1:200); anti-collagenIV (Chemicon, AB756P; 1:100) followed by goat anti-abbit IgG H+L secondary antibody (Alexa Fluor-488, Molecular Probes; 1:500) and mounted with Vectashield mountant containing DAPI. Images were acquired using an epifluorescence microscope (Carl Zeiss). For immunohistochemistry, EBs were fixed in 4% paraformaldehyde, dehydrated and embedded in paraffin wax using standard methods. Dewaxed sections were processed for antigen retrieval by boiling for 20 min in Dako antigen retrieval solution and washed in PBS-T (0.02%) for 10 min. Slides were blocked with either 5% skimmed milk or 5% goat serum for 1 hour, then incubated with primary antibody overnight at 4°C at 1:400 dilution. Antibodies included anti-laminin, anti-collagenIV (as before) and anti-Dab2 (BD Transduction 610464). Sections were washed three times in PBS-T for 5 min. Blocking of endogenous peroxidase, incubation with peroxidase-labelled polymer and detection with DAB + chromagen were performed according to the manufacturer's instructions (DAKO kit). Sections were counterstained with haematoxylin using standard methods.

### Basement membrane staining and whole-mount in situ hybridisation analysis of embryos

E6.5 embryos were fixed in 4% PFA/PBS, washed in 0.1% TritonX/PBS and permeabilised in 0.5% TritonX/PBS. Following three washes in 0.1% TritonX/PBS, embryos were blocked in 5% FBS, 0.2% BSA, 0.1% TritonX - PBS and incubated overnight in primary rabbit polyclonal anti-Collagen IV (Chemicon AB756; 1:100). Embryos were washed extensively with 0.1% TritonX/PBS and then incubated in anti-rabbit Alexa-Fluor 488 secondary antibody (Invitrogen A11034; 1:200). Embryos were further washed extensively with 0.1%TritonX/PBS, immersed in Vectashield mounting media containing DAPI and imaged using laser scanning confocal microscopy (Zeiss LSM 710 confocal microscope). Whole mount *in situ *hybridisation was performed according to standard procedures [88] using a probe for *Oct4 *[5]. The experimental protocols described in this report have been approved by the Ethical Review Committee of the University of Oxford.

## Authors' contributions

IC carried out Q-PCR, Western blot analysis and microscopy experiments and wrote the manuscript. CAB conceived the project, conducted the microarray experiments, annotated the gene list and conducted early Q-PCR. JMT provided advice about the experimental design and conducted bioinformatics analysis. EKB assisted with Figure design and edited the manuscript. EJR provided ES cell lines, supervised the project, provided grant support and finalised the manuscript. All authors read and approved the final manuscript.

## Supplementary Material

Additional file 1 **Full list of genes mis-regulated in ES array**. Data is divided into phase 1 and phase 2, as described in the materials and methods. Click here for file

Additional file 2 **Full list of genes mis-regulated in EB array**. Click here for file

Additional file 3 **Laminin and Collagen IV staining of suspension EBs and EB outgrowths**. **A**. Day 4 wild-type and Smad4 null EBs grown for 2 days on fibronectin-coated (25 μg/ml) dishes. Smad4 mutant EBs express increased levels of Collagen IV **B**. Cryosections of day4, day7 and day10 suspension EBs stained for laminin. Smad4 mutant EBs display increased basement membrane deposition beneath the outer endoderm layer. Click here for file

Additional file 4 **Primers**. Indicated is the gene name, forward and reverse primer sequence, expected product size and annealing temperature used for Q-PCR. Click here for file
