# Supplementary figures and images for: Smad4-dependent pathways control basement membrane deposition and endodermal cell migration at early stages of mouse development
Source: BMC Dev Biol. 2009 Oct 22;9:54. doi: 10.1186/1471-213X-9-54 (PMC2773778; doi:10.1186/1471-213X-9-54)

A.

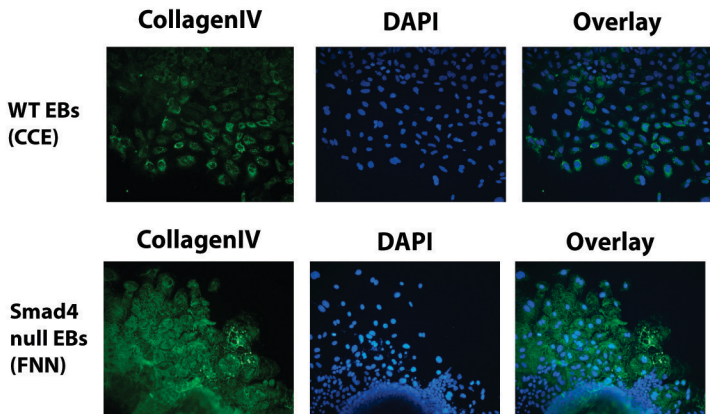

B.

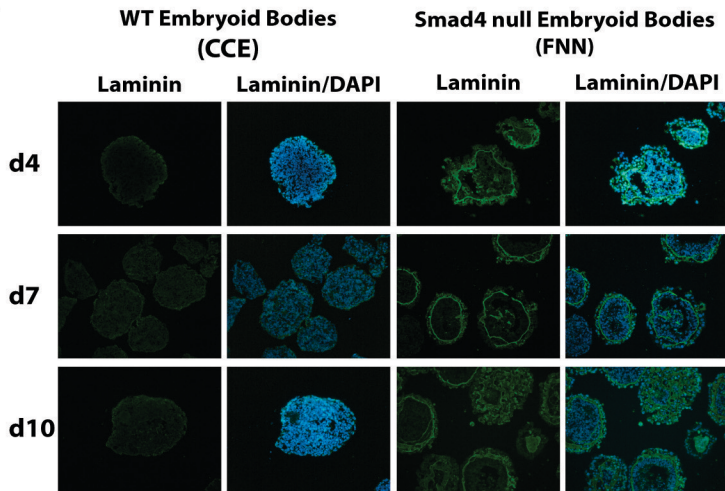

Supplement: Additional file 3 — Laminin and Collagen IV staining of suspension EBs and EB outgrowths. A. Day 4 wild-type and Smad4 null EBs grown for 2 days on fibronectin-coated (25 μg/ml) dishes. Smad4 mutant EBs express increased levels of Collagen IV B. Cryosections of day4, day7 and day10 suspension EBs stained for laminin. Smad4 mutant EBs display increased basement membrane deposition beneath the outer endoderm layer. [file 1471-213X-9-54-S3.PDF]
