# Supplementary material for: Smad4-dependent pathways control basement membrane deposition and endodermal cell migration at early stages of mouse development
Source: BMC Dev Biol. 2009 Oct 22;9:54. doi: 10.1186/1471-213X-9-54 (PMC2773778; doi:10.1186/1471-213X-9-54)
Supplement: Additional file 4 — Primers. Indicated is the gene name, forward and reverse primer sequence, expected product size and annealing temperature used for Q-PCR. [file 1471-213X-9-54-S4.DOC]

**Additional File 4 -** *Primers*

| **Gene Name** | **Primer Seq. Forward** | **Primer Seq. Reverse** | **Product Size** | **Tm (^o^C)** |
| --- | --- | --- | --- | --- |
| Tuba3a | GCGGCGTCTGAGCGGGTCTCC | GGTTCCCGTGCGCACCTCATCC | 277 bp | 60 |
| Gsc | AAACGCCGAGAAGTGGAACAAG | AAGGCAGGGTGTGTGCAAGTAG | 177 bp | 56 |
| Sp5 | CCATCGAGGTAGCTGACAAAGAGT | TGTAGCTCTGCGTGGAGCTGAGA | 393 bp | 56 |
| Tcl1 | TGGCCTCACTAGAACAAGAGG | CTCGGTCAAGGATGGAAGC | 180 bp | 55 |
| Dpp4 | AGGATCACATCGACAGGAGAA | TTGTTTGGAGACCACCACAG | 110 bp | 56 |
| En1 | ACACAACCCTGCGATCCTACTC | CGCTTGTCTTCCTTCTCGTTCT | 180 bp | 56 |
| Fgf5 | TTGCGACCCAGGAGCTTCCT | CTACGCCTCTTTATTGCAGC | 209 bp | 55 |
| Tbx3 | TTATTTCCAGGTCAGGAGATGGC | GGTCGTTTGAACCAAGTCCCTC | 397 bp | 56 |
| Lhx1 | CCCAGCTTTCCCGAATCCT | GCGGGACGTAAATAAATAAAATGG | 74 bp | 55 |
| MixL1 | ACTTTCCAGCTCTTTCAAGAGCC | ATTGTGTACTCCCCAACTTTCCC | 487 bp | 55 |
| Fgf8 | CATGGCCTTTACCCGCAAG | CGGGTAGTTGAGGAACTCGAAG | 142 bp | 55 |
| Esrrb | CAGGCAAGGATGACAGACG | GAGACAGCACGAAGGACTGC | 178 bp | 55 |
| Dppa4 | TGCCCCAAGTGTGTTCATAA | TTCTGGATGAGAAAGGCACC | 186 bp | 55 |
| Dnmt3l | TTCATGGACAATGTGCTGCT | TGACTTGGGCTTGCAGATAC | 199 bp | 56 |
| Mmp9 | GGCGTTAGGGACAGAAATGTTG | TCATCTCACCTGGAGGACACAGTC | 229 bp | 56 |
| Cd44 | CCAACACCTCCCACTATGAC | TATACTCGCCCTTCTTGCTG | 159 bp | 56 |
| Ndp52L1 | CATGAGCAGCTACAGAGGAAGCA | GTGCCTCAGATTCACTGTGTAGCTAA | 337 bp | 56 |
| Magea8 | CCTGCTGTGATTGGAACACTATCC | GACTCTGGGGAGGACTTGGTATTC | 217 bp | 56 |
| Eomes | TGTTTTCGTGGAAGTGGTTCTGGC | AGGTCTGAGTCTTGGAAGGTTCATTC | 323 bp | 58 |
| Brachyury | AACTTTCCTCCATGTGCTGAGAC | TGACTTCCCAACACAAAAAGCT | 533 bp | 55 |
| Foxa2 | TGGCTGCAGACACTTCCTACT | CAACATCAGTACAACCCTCTGGT | 487 bp | 55 |
| Aire | CAAGGTAGGGGACGACTCTGC | GAGGGAGCCAATGCTGTGC | 201 bp | 62 |
| Gdf1 | TGGCTTCCTAGCCAACTTCTGC | AGGACCACGTTGTCACTATTGTCG | 210 bp | 58 |
| Lefty1 | TGTGTGTGCTCTTTGCTTCC | GGGGATTCTGTCCTTGGTTT | 180 bp | 58 |
| Lefty2 | CAGCCAGAATTTTCGAGAGGT | CAGTGCGATTGGAGCCATC | 175 bp | 58 |
| Id1 | GTGAGCAAGGTGGAGATCCTGC | CCAAAGTCTCTGGAGGCTGAAAGG | 398 bp | 58 |
| Id2 | GCATCCCCCAGAACAAGAAGGT | CTCTCATAAATAACGGTATCACAG | 474 bp | 55 |
| Id3 | GGTGCGCGGCTGGTACGA | CAGGCCACCCAAGTTCAGTCC | 546 bp | 60 |
| Gata6 | AGACATAACATTCCTTCGATGCG | TTCCAAGTGACCTCAGATCAGC | 504 bp | 55 |
| Dab2 | GAGCGAGGACAGAGGTCAAC | AAAGGACATTCCCAGTGACG | 521 bp | 60 |
| Pramel7 | GTGAGGAATGAAGTATTGACCGT | CCTCTCTCTCATCCTGCATATCT | 313 bp | 60 |
| Pramel4 | GAAAACGCTGCTGCAACATA | GAGCAGTCGGGTGTTCCTAC | 308 bp | 60 |
| Igf2 | AGGGGAGCTTGTTGACACG | GGGTATCTGGGGGAAGTCGTC | 225 bp | 60 |
| H19 | TACCCCGGGATGACTTCATC | TATCTCCGGGACTCCAAACC | 186 bp | 58 |
| Decorin | CAACCTTGCTAGACCTGCAAA | CTGGAGAGTTCTGGGCATTTTT | 212 bp | 60 |
| Mmp14 | AGTGACAGGCAAGGCTGATTT | AGGGGTGTAATTCTGAATGCAG | 175 bp | 58 |
| Hic-5 | GCTTCAGGAACTTAATGCCA | GAAGTCAGAGAGTGAGGCCA | 217 bp | 58 |
| Zscan4 | GTTCAAACGAGTCTCTGACCGC | CGATGGTAAGTGGATGATTGGC | 195 bp | 56 |
| Hprt | GCTGGTGAAAAGGACCTCT | CACAGGACTAGAACACCTGC | 249 bp | 55-62 |
